# Astrocyte Signature in Alzheimer’s Disease Continuum through a Multi-PET Tracer Imaging Perspective

**DOI:** 10.3390/cells12111469

**Published:** 2023-05-24

**Authors:** Igor C. Fontana, Miriam Scarpa, Mona-Lisa Malarte, Filipa M. Rocha, Sira Ausellé-Bosch, Marina Bluma, Marco Bucci, Konstantinos Chiotis, Amit Kumar, Agneta Nordberg

**Affiliations:** 1 Division of Clinical Geriatrics, Center for Alzheimer Research, Department of Neurobiology, Care Sciences and Society, Karolinska Institutet, 141 52 Stockholm, Sweden; 2 Instituto de Ciência Biomédicas Abel Salazar da Universidade do Porto, 4050-313 Porto, Portugal; 3 Faculdade de Engenharia, Universidade do Porto, 4200-465 Porto, Portugal; 4 Faculty of Health and Life Sciences, Pompeu Fabra University, 08003 Barcelona, Spain; 5 Theme Inflammation and Aging, Karolinska University Hospital, 141 57 Stockholm, Sweden

**Keywords:** reactive astrogliosis, positron emission tomography, Alzheimer’s disease, L-deprenyl, BU99008, SMBT-1, GFAP

## Abstract

Reactive astrogliosis is an early event in the continuum of Alzheimer’s disease (AD). Current advances in positron emission tomography (PET) imaging provide ways of assessing reactive astrogliosis in the living brain. In this review, we revisit clinical PET imaging and in vitro findings using the multi-tracer approach, and point out that reactive astrogliosis precedes the deposition of Aβ plaques, tau pathology, and neurodegeneration in AD. Furthermore, considering the current view of reactive astrogliosis heterogeneity—more than one subtype of astrocyte involved—in AD, we discuss how astrocytic body fluid biomarkers might fit into trajectories different from that of astrocytic PET imaging. Future research focusing on the development of innovative astrocytic PET radiotracers and fluid biomarkers may provide further insights into the heterogeneity of reactive astrogliosis and improve the detection of AD in its early stages.

## 1. Introduction

Based on the latest consensus, the definition of Alzheimer’s disease (AD) comprises clinical phenotypes and a biological construct composed of amyloid-β (Aβ) deposition, pathological tau, and neurodegeneration—the AT(N) research framework [1,2]. In an attempt to improve AD detection, the spotlight has now turned to the early stages of the disease (i.e., prior to the deposition of Aβ plaques) [3]. In this regard, the supplementation of a new category of the AT(N) framework has been suggested—the ATX(N)—in which “X” represents potential innovative candidate biomarkers (body fluids or imaging) that could reveal early pathophysiological changes in the AD continuum [3]. A promising target for the early detection of AD, which for many years has been neglected due to neurocentric approaches, is the astrocyte [4]. Astrocytes are the most abundant glial cells in the human brain, playing crucial roles in brain energetic metabolism, neurotransmitter recycling and release, and redox balance, among other functions (reviewed in Semyanov and Verkhratsky, 2021 [5]). In response to pathological insults, astrocytes become reactive, a phenomenon termed reactive astrogliosis [6]. According to a recent consensus, reactive astrogliosis is defined as “*the process whereby, in response to pathology, astrocytes engage in molecularly defined programs involving changes in transcriptional regulation, as well as biochemical, morphological, metabolic, and physiological remodeling, which ultimately result in gain of new function(s) or loss or upregulation of homeostatic ones*.” [6].

Biological changes in AD, including reactive astrogliosis, can be detected in vivo by measuring fluid biomarkers from the cerebrospinal fluid (CSF) and plasma, or using brain imaging tools. Among the imaging modalities, only positron emission tomography (PET) allows for a spatiotemporal investigation of pathophysiological processes in vivo [7]. ^11^C-Deuterium-L-deprenyl (^11^C-DED) (often synthesized without deuterium as ^11^C-L-deprenyl) is a well-established PET radiotracer used in AD research that targets monoamine oxidase B (MAO-B) for the imaging of astrocytes [4,8,9,10,11,12,13,14,15,16,17,18,19,20,21]. Recently, ^18^F-SMBT-1 and ^11^C-BU99008, which bind to MAO-B and I2-imidazoline binding site (I_2_BS), respectively, have been developed as alternative tools to assess reactive astrogliosis in AD brains [9,22,23] (Table 1). The characteristics of each of these three PET radiotracers are discussed below.

To define how reactive astrogliosis fits in the ATX(N) framework, it is crucial to investigate the relationship between this phenomenon and Aβ deposition, tau pathology, and neurodegeneration in the AD continuum. In this context, the concept of multi-tracer studies has been designed to explore the associations between different AD pathophysiological hallmarks. In vitro autoradiography studies in postmortem human brain are a valuable complement since they provide a platform to validate the multi-tracer approach, and to investigate in detail the binding properties and associations among different PET radiotracers. In this review, we discuss how the translational multi-tracer approach, in vivo and in vitro, with the different astrocytic PET radiotracers DED, SMBT-1, and BU99008, provide important new information to understand the complex signature of reactive astrogliosis, including heterogeneity and possible associations with AT(N)-biomarkers in AD. Our main goal is to highlight the significance of reactive astrogliosis as a target for the early, presymptomatic detection of AD and its progression. Furthermore, we provide initial hints on how molecular/functional changes assessed by astrocytic PET tracers could have a trajectory different from fluid glial fibrillary acidic protein (GFAP) levels in the early stages of AD.

## 2. Reactive Astrogliosis, We Can See You!

For decades, an increased level of GFAP, evaluated by immunohistochemistry, has been pointed out as a universal marker of reactive astrogliosis [28]. However, recent transcriptomic analyses indicate that sole reliance on GFAP is unlikely to define the broad range of reactive astrogliosis in AD [29]. Therefore, different tools to detect reactive astrogliosis are required to depict both the heterogeneity of astrocytes and their role in AD brains [29,30]. A wide range of proteins is differentially expressed in reactive astrogliosis, including receptors, transporters, and enzymes, with great potential to become surrogate biomarkers for developing PET radiotracers to detect heterogenous populations of astrocytes in the brain in vivo [20,31]. Among these, MAO-B, an enzyme present in the outer membrane of mitochondria, was initially shown to localize in GFAP-positive astrocytes [32]. Remarkably, additional studies indicated that the overexpression of MAO-B in response to Aβ pathology had no or limited correlation with increased GFAP levels, and instead GFAP levels increase at later stages [33]. These observations lead to the possibility that increases in MAO-B and GFAP potentially reflect two mechanisms of reactive astrogliosis and perhaps different populations of astrocytes. Thus, targeting MAO-B and GFAP overexpression may offer the possibility of exploring the heterogeneity of reactive astrogliosis in AD. These findings fostered the development of ^11^C-DED, which is the gold-standard PET radiotracer for imaging reactive astrogliosis in the living brain. Clinical PET imaging with ^11^C-DED showed reactive astrogliosis in different brain diseases such as epilepsy, Creutzfeldt–Jakob disease, amyotrophic lateral sclerosis, and AD [9,34,35,36]. In the context of AD, ^11^C-DED PET has provided insightful information on early pathological changes in the continuum of the disease, which we describe in detail in the next section.

Recently, ^18^F-SMBT-1, a fluorine-18 radiolabeled PET radiotracer selective for MAO-B, was developed [37]. The human evaluation of ^18^F-SMBT-1 highlighted its reversible binding properties with high blood–brain barrier (BBB) permeability and low non-specific binding. In addition, ^18^F-SMBT-1 binding was most pronounced in basal ganglia and cortical regions, with lower detection in the cerebellum and white matter, in accordance with the regional expression of MAO-B in the human brain. Therefore, ^18^F-SMBT-1 stands out as an additional tool for detecting reactive astrogliosis in AD brains [25]. The PET radiotracer ^11^C-BU99008 was developed to be a non-MAO-B selective tool for imaging reactive astrogliosis [23,27]. This PET radiotracer binds with high affinity to I_2_BS in the outer mitochondrial membrane [38]. I_2_BS is expressed in both neurons and astrocytes [39], but similar to MAO-B, the overexpression of I_2_BS is mostly observed in astrocytes (assessed by co-localization with GFAP immunoreactivity) and associated with pathology [40]. ^11^C-BU99008 is characterized by its good BBB penetration, reversible kinetics, great specificity and selectivity, and regional distribution, according to the expression of I_2_BS in the human brain [27,38].

Recently, we have provided a very detailed comparison between DED, BU99008, and SMBT-1 binding in vitro using postmortem AD and cognitively normal (CN) brains. Initially, we demonstrated that ^3^H-DED and ^3^H-BU99008 have similar regional distribution with higher binding in the hippocampus and frontal cortex of AD brains compared to CN ones [41]. However, ^3^H-DED and ^3^H-BU99008 differ in their number of binding sites in CN and AD brains [41]. In contrast to ^3^H-DED, which detects a single binding site, ^3^H-BU99008 has multiple binding sites with a wide range of affinities [41]. In the same context, SMBT-1 appears to have a binding pattern similar to ^3^H-DED; however, it also displaces ^3^H-BU99008 at multiple binding sites [21]. This could be explained by the presence of I_2_BS in the substrate entrance channel of MAO-B [42], whereby the binding of SMBT-1 to MAO-B potentially causes steric hinderance affecting the binding of ^3^H-BU99008 to the I_2_BS binding site. Therefore, even though PET radiotracers such as BU99008 are developed to selectively target I_2_BS, it might not be possible to discriminate between changes in MAO-B and I_2_BS in AD brains.

## 3. Amyloid-β Pathology—A Consequence or Trigger of Reactive Astrogliosis?

**Figure 1:**
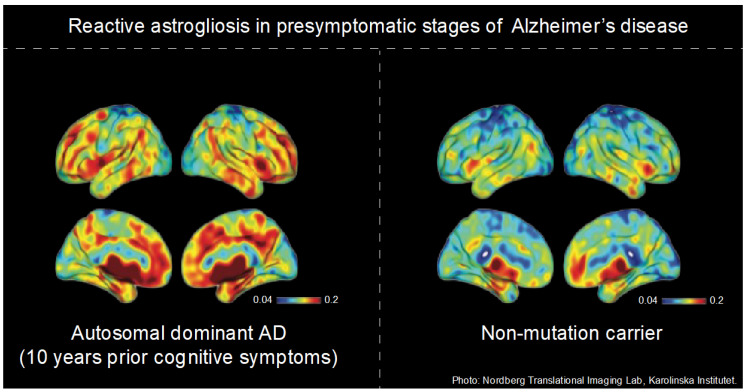
^11^C-L-Deprenyl binding in a presymptomatic autosomal dominant Alzheimer’s disease (ADAD) carrier. In ADAD mutation carriers, increased ^11^C-L-Deprenyl binding compared to mutation non-carriers is evident approximately 10 years before the presymptomatic stages. Figure credit: Nordberg Translational Imaging Lab, Karolinska Institutet.

The Aβ peptide (ranging from 36 to 43 amino acids) is a product of the consecutive cleavage of a transmembrane protein termed amyloid precursor protein. Once released in the form of monomers, Aβ tends to aggregate into soluble oligomers, protofibrils, and fibrils and then forms deposits of insoluble Aβ plaques [43]. The deposition of insoluble Aβ can be detected with ^11^C-Pittsburgh compound B (PiB) [44], ^18^F-NAV4694 [45], and the FDA-approved ^18^F-Florbetaben [46], ^18^F-Florbetapir [47], and ^18^F-Flutemetamol [48]. Clinical multi-tracer PET studies using amyloid- and astrocyte-PET radiotracers have established higher ^11^C-DED binding in the frontal and parietal cortices—regions enriched in astrocytes—of mildly cognitive-impaired (MCI, also referred to as prodromal AD [2]) and sporadic AD patients (sAD) compared to cognitive normal (CN) individuals [8,9,12]. Interestingly, increased ^11^C-DED binding was more evident in MCI patients who were ^11^C-PiB-positive (i.e., have a considerable amount of Aβ plaque deposits or are amyloid positive, A+), indicating that reactive astrogliosis is associated with Aβ pathology in MCI A+ individuals (prodromal AD) [9]. These findings were crucial to confirm previous immunohistochemical reports indicating a link between reactive astrogliosis and amyloidosis in AD [28]. However, these initial studies could not clarify whether reactive astrogliosis is a response to, or an instigator of, Aβ pathology in the early stages of AD. In this context, clinical multi-PET tracer studies in autosomal dominant AD (ADAD) cases offer the possibility of investigating whether reactive astrogliosis precedes Aβ deposition. The carriers of these genetic mutations develop AD symptoms onset at a mutation-specific age, allowing the preclinical trajectories of different biomarkers to be tracked at an individual level [12,14]. Remarkably, increased ^11^C-DED binding was observed in the presymptomatic stages of ADAD approximately 10 years before the expected onset of clinical symptoms, followed by a decline across the disease continuum (Figure 1) [12]. Conversely, Aβ deposition progressively increased from early to advanced stages of the disease, until it reached a plateau [12].

The relationship between Aβ pathology and reactive astrogliosis has also been assessed with ^18^F-SMBT-1 and ^11^C-BU99008. Cross-sectional studies using ^18^F-SMBT-1 demonstrated higher binding in multiple regions in AD brains compared to CN brains, including in the posterior cingulate gyrus, supramarginal gyrus, and lateral occipital lobe, and, to a lesser extent, in the hippocampus and globus pallidus [22]. In a multi-tracer study comparing CN, MCI, and sAD cases, reactive astrogliosis, assessed by ^11^C-BU99008 binding, was evident in frontal, parietal, and occipital areas of Aβ-positive patients, where it positively correlated with Aβ plaque load measured by ^18^F-Florbetaben PET [49]. Interestingly, ^11^C-BU99008 binding was more pronounced in the cingulate, frontal, and temporal cortices of MCI compared to AD individuals [49].

As noted above, ^11^CL-deprenyl binding is most prominent in the early stages of AD, prior to the deposition of Aβ plaques. Despite some limitations (including total number of AD subjects and mixing Aβ-positive and Aβ-negative individuals), findings on ^11^C-BU99008 and ^18^F-SMBT-1 provided initial hints that these PET-radiotracers follow similar patterns and could serve as surrogate markers of reactive astrogliosis. We suggest that pre-plaque soluble Aβ forms could be the main culprits that trigger reactive astrogliosis, which, in turn, may lead to the formation and spreading of Aβ in AD [50]. In a recent review article, we put forward a hypothesis in which we reported that the overexpression of α7 nicotinic acetylcholine receptors in reactive astrocytes may promote the formation of astrocytic Aβ plaques [51].

In vitro autoradiography studies using sAD and CN postmortem brain tissue support clinical research findings, pointing towards region-specific associations between reactive astrogliosis and Aβ pathology [10,18]. Comparative binding studies in sAD and CN tissues found that ^3^H-DED had higher binding throughout the whole sAD hippocampus, whereas ^3^H-PiB had low and uniform binding in this sAD brain region [10,18]. Conversely, in the frontal cortex, ^3^H-PiB binding was high in all layers, while ^3^H-DED showed a different pattern, with more pronounced binding in the superficial laminar sections [10,18]. Remarkably, GFAP levels determined by immunohistochemistry did not follow the same pattern as those of ^3^H-DED binding in cortical regions. Instead, GFAP-positive cells were found uniformly distributed across the superficial cortical layers as well as in the cortical layer bordering the white matter and were concentrated around Aβ plaques in deeper layers [10]. The differences in the overexpression of GFAP and MAO-B (the latter demonstrated by ^3^H-DED) observed in these results, together with studies in mouse models of disease [33], led to the hypothesis that increases in GFAP or MAO-B in AD reflect distinct subtypes of astrocytes displaying different responses to AD pathology, in a region-specific manner [10,13].

## 4. Pathological Tau and Reactive Astrogliosis

Tau is the main microtubule-associated protein in neuronal cells. The accumulation of hyperphosphorylated tau leads to the formation of neurofibrillary tangles, a key pathological hallmark of AD [52]. PET radiotracers that image tau pathology can be divided into first (^18^F-AV-1451 (Flortaucipir), ^18^F-THK5317, ^18^F-THK5117, and ^11^C-PBB3) [53] and second generation (^18^F-MK6240, ^18^F-PI2620, ^18^F-RO948, ^18^F-PM-PBB3, ^18^F-APN-1607, ^18^F-GTP1, and ^18^F-JNJ31) [54]. To date, only ^18^F-Flortaucipir has been FDA approved to target pathological tau for clinical diagnosis [55], but other tau tracers have been used in clinical research settings (following the required ethical approval). To our knowledge, only one clinical in vivo PET study has been conducted assessing astrocytic and tau PET radiotracers in the same set of individuals [22]. The authors demonstrated that ^18^F-SMBT-1 binding positively correlates with ^18^F-MK6240 in the temporoparietal cortex, and the supramarginal and posterior cingulate of patients across the AD continuum. This points towards a brain-region-specific association between reactive astrogliosis and tau pathology, although the degree of correlation is less than that of ^18^F-SMBT-1 and amyloid-PET assessed by ^18^F-NAV4694 in the same brain regions.

When our group assessed ^3^H-THK5117 and ^3^H-L-deprenyl binding in postmortem AD brains at advanced disease stages, we observed similar laminar distribution patterns [15]. Both ^3^H-THK5117 and ^3^H-L-deprenyl showed high binding in the superficial and deep layers of temporal cortices and diffuse binding throughout the middle frontal gyrus [15]. These observations are comparable with the immunostaining pattern of the GFAP antibody and AT8 antibody, which is specific for the phosphorylated form of tau [15].

In ADAD carriers of the ΔE9 mutation in the presenilin 1 gene (PSEN1ΔE9) or the Arctic mutation in the amyloid-beta precursor protein (APParc), ^3^H-THK5117 and ^3^H-DED displayed a similar laminar distribution (bilayer pattern) throughout the cortex, which was corroborated by GFAP and AT8 immunostaining [19]. Interestingly, a significant positive correlation between ^3^H-DED and ^3^H-THK5117 binding could only be observed in APParc brains, indicating there might be a closer relationship between tau and reactive astrocytosis in APParc brains than in PSEN1ΔE9 brains [19]. The APParc mutation is mostly characterized by fewer dense-core Aβ plaque deposits and more soluble Aβ forms than the PSEN1DE9 mutation. In this context, the stronger relationship between reactive astrogliosis and tau deposits in the APParc brains, compared to sAD and PSEN1ΔE9 brains [19], could be accounted for by higher exposure to soluble Aβ oligomers—the suggested top culprits of toxicity in AD pathophysiology—and by the fact that Aβ plaque pathology is not a requirement to trigger tau pathology and reactive astrogliosis.

## 5. Neurodegeneration and Reactive Astrogliosis: Two Separate Phenomena?

The fluorine-18 radiolabeled analogue of glucose—^18^F-2-fluoro-2-deoxy-d-glucose (^18^F-FDG)—PET radiotracer is a gold-standard biomarker to assess neurodegeneration in vivo. ^18^F-FDG PET measures the rate of transport and trapping of the tracer, which has traditionally been attributed to neuronal glucose uptake, thus reflecting neuronal activity [56]. The first multi-tracer study that explored a possible link between neurodegeneration (evaluated with ^18^F-FDG) and reactive astrogliosis (assessed with ^11^C-DED) demonstrated no relationship between these two phenomena in either MCI or sAD individuals [9]. In a cross-sectional assessment of ADAD (presymptomatic and symptomatic), MCI, and sAD subjects, ^18^F-FDG hypometabolism was mostly evident in the symptomatic/later stages, opposite to the trend of ^11^C-DED and ^11^C-PiB binding (higher since earlier stages) [12]. A subsequent study using a longitudinal approach demonstrated that ^11^C-DED binding had significant positive associations with ^18^F-FDG uptake in presymptomatic ADAD individuals across all brain regions of interest except in the hippocampus and frontal cortex—regions commonly affected by Aβ pathology—where no significant correlations were found [16]. These findings corroborate the view of astrocytes being contributors to the ^18^F-FDG signal. In regions less affected by AD pathology, astrocytes may act in a compensatory way to maintain brain energetic homeostasis [57,58].

An exploratory analysis of ^11^C-BU99008 in CN, MCI, and AD individuals demonstrated, on a voxel-wise basis, a correlation between reduced ^18^F-FDG uptake and reduced ^11^C-BU99008 binding in the parietal, frontal, and temporal lobes of ^18^F-Florbetaben-positive subjects [49]. These findings were not statistically confirmed with a regional-based correlation analysis, thus blurring any conclusions regarding associations between reactive astrogliosis, assessed by ^11^C-BU99008, and neurodegeneration [49]. Notably, no studies correlating SMBT-1 with neurodegeneration have been conducted so far.

Since neuronal and astrocytic ^18^F-FDG uptake cannot be separately measured [59], alternative tools are required to investigate neurodegeneration in vivo. Moreover, FDG-PET is unlikely to reflect changes at the synaptic level (density, function, and structure). The recently developed novel PET radiotracer UCB-J, which targets the synaptic vesicle 2A (SV2A) as an index of synaptic density [60], might help define the potential link between reactive astrogliosis and neurodegeneration in AD [61]. Ongoing studies from our group have also shown that ^3^H-UCB-J can target SV2A with high specificity in AD and CN brains [62]. Alternatively, the relationship between reactive astrogliosis and neurodegeneration can be explored using other imaging modalities such as magnetic resonance imaging (MRI). We have demonstrated a positive correlation between cortical microstructure and ^11^C-DED PET binding in ADAD carriers [17], indicating that microstructural MRI changes may reflect reactive astrogliosis in the brain of these individuals. Furthermore, we have also reported that in the parahippocampus of MCI Aβ+ individuals, increased ^11^C-DED binding correlated with grey matter (GM) density loss—a surrogate marker of brain atrophy assessed by MRI [11]. In this context, reactive astrogliosis might play a role in the structural brain changes in sAD. Overall, even though associations between ^11^C-DED and ^18^F-FDG uptake could only be observed in presymptomatic ADAD individuals, one cannot rule out that reactive astrogliosis may impair brain metabolism in the early stages of the disease, triggering downstream signaling pathways that might be associated with neurodegeneration in the AD dementia phase.

## 6. Relationship between Imaging and Fluid Astrocytic Biomarkers in AD

Apart from PET imaging, several CSF and blood biomarkers have been developed to track reactive astrogliosis heterogeneity. A few examples are GFAP, S100 calcium-binding protein B, aquaporin-4, and chitinase-3-like protein 1 (YKL-40) [20]. In fact, CSF levels of YKL-40 are associated with tau-PET [63], while GFAP (mainly plasma) correlated better with Aβ-PET [63,64,65]. This could indicate that (1) increases in CSF YKL-40 and plasma GFAP reflect distinct populations of astrocytes with unique responses to different AD-related pathologies, and (2) plasma GFAP could be a marker of Aβ pathology. In a cohort of Karolinska memory clinic patients, plasma GFAP levels correlated significantly with PET-detected amyloid deposits, but not with measure of pTau in CSF, whereas plasma pTau isoforms were associated with both measures of amyloid and tau pathology [66]. Nevertheless, the biological interpretation of these findings is still blurred and leaves a few questions. For instance, do higher GFAP levels in plasma reflect astrocytic responses originating in the central nervous system only? Or does peripheral GFAP also contribute to these changes (do chondrocytes, in a subpopulation of quiescent liver stellate cells, myoepithelial cells, and fibroblasts also express GFAP? [67])? Finally, most importantly, does plasma/CSF GFAP provide the same information as ^11^C-DED PET?

When tracking reactive astrogliosis in ADAD and sAD individuals compared to healthy controls, we have observed a negative correlation between plasma GFAP levels and ^11^C-L-deprenyl PET binding in vivo [68]. Furthermore, in a recent cross-sectional study on ADAD mutation carriers, plasma GFAP elevations emerge a decade before symptoms onset, prior to neurodegeneration, but after Aβ deposition commences [69,70]. In keeping with this, plasma GFAP seems to follow a different trajectory to ^11^C-DED PET, which increases before the appearance of Aβ plaques. Hence, it might be possible that astrocytic PET imaging and plasma GFAP concentrations reflect independent aspects of reactive astrogliosis in AD, depicting different stages or subtypes of astrocytes. We hypothesize that ^11^C-DED PET may illustrate a scenario in which ‘Pre-plaque stage’ soluble Aβ species trigger early molecular/functional changes in reactive astrocytes (characterized by MAO-B overexpression) and, with disease progression, Aβ aggregates into insoluble plaques (Aβ-plaque stage) and reactive astrocytes remodel their cytoskeleton (i.e., increase GFAP expression) and become hypertrophic. Why GFAP “leaks” into the blood is a question which needs further investigations.

## 7. Concluding Remarks

PET imaging plays a fundamental role not only in defining the biological construct of AD, but also in exploring the interplay between pathological hallmarks in AD progression. In this review, we revisited clinical biomarker findings and in vitro autoradiography studies providing spatiotemporal associations between reactive astrogliosis and Aβ, tau pathology, and neurodegeneration in order to evaluate the possibility that astrocytes contribute to the X in the ATX(N) research framework.

**Figure 2:**
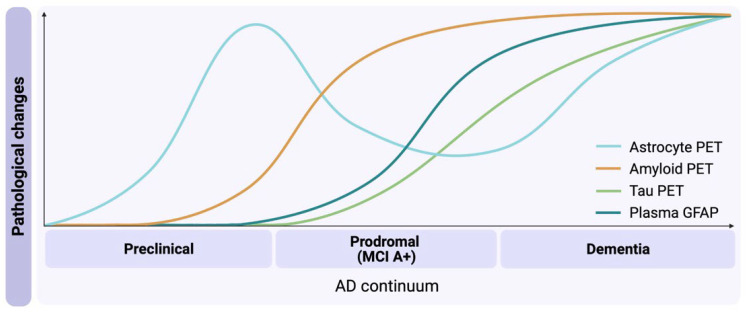
Reactive astrogliosis in the Alzheimer’s disease (AD) continuum. The light blue curve illustrates increased astrocyte PET signal in preclinical AD assessed by ^11^C-DED (or the version synthesized without deuterium, L-Deprenyl), a selective, irreversible monoamine oxidase type B, MAO-B, inhibitor. This is the first sign of reactive astrogliosis (i.e., the first wave). Following Aβ plaque deposition (orange curve), the astrocyte PET curve declines. As a consequence of Aβ deposition, GFAP levels increase in plasma (teal curve); however, further studies are required to define whether this phenomenon reflects reactive astrogliosis (a different state than that characterized by MAO-B overexpression as per ^11^C-L-Deprenyl PET signal) or changes in peripheral cells that also express GFAP. Subsequently, tau pathology (green curve) and a second wave of reactive astrogliosis assessed by ^11^C-L-Deprenyl can be detected, characterizing the later stages of AD (i.e., AD dementia). MCI A+, mild cognitive impairment with positive amyloid.

The spatiotemporal associations between reactive astrogliosis and other AD biomarkers seem to be complex. To shed light on this intricate relationship, we recently proposed a ‘Two wave model of reactive astrogliosis’ in the AD continuum, based on our multi-tracer clinical/translational studies ranging from in vitro to in vivo PET brain imaging [4]. In the early stages of AD, reactive astrogliosis is characterized by the increased expression of MAO-B (first wave of astrogliosis), which has a positive correlation with early Aβ deposition. The interplay between astrocytes and Aβ pathology at this stage is multifaceted. Since reactive astrogliosis was shown to precede significant build-up of Aβ plaque [12,23], we suggest that astrocytes could promote plaque formation, and/or that pre-plaque soluble Aβ (not detected with the current PET radiotracers) may trigger astrocyte dysfunction, for example, the abnormal production of reactive oxygen species, abnormal glutamate release, and hyperactivation of extrasynaptic NMDA receptors [50]. Across the continuum of the disease, as the prodromal stage progresses, in brain regions of advanced Aβ pathology (i.e., high amyloid-PET), astrocytes may become dystrophic, marking the end of the first wave of astrogliosis (i.e., ^11^C-DED PET labeling declines) and the beginning of tau pathology detection (Figure 2).

Although the mechanisms linking these three pathological hallmarks remain to be elucidated, it is possible that tau pathology could be exacerbated in the crosstalk between reactive astrocytes and activated microglial in response to soluble Aβ oligomers [71,72]. Positive associations between reactive astrogliosis and tau pathology, as well as with amyloid load at advanced stages of AD, can also be explained by the presence of resilient astrocytes (second wave of astrogliosis), which are resistant to pathological insults and cell death but functionally dormant. The involvement of astrocytes in neurodegeneration in later stages of AD remains unclear. Nevertheless, the positive correlation between ^18^F-FDG levels and ^11^C-DED binding in the presymptomatic phase of ADAD suggests that hyperactivity in brain energetic metabolism associated with reactive astrogliosis could be an initial trigger for neurodegeneration [57,58].

As the field of biomarker research moves at a fast pace, the combination of innovative astrocytic PET radiotracers and fluid biomarkers will provide further insights into the complexity of mechanisms associated with reactive astrogliosis across the continuum of AD, potentially shedding light on new tools for detecting the disease in the early stages.

## Figures and Tables

**Table 1:** PET radiotracers for imaging reactive astrocytes in Alzheimer’s disease.

PET Radiotracer	Target *	Ligand Characteristics
^11^C-Deuterium-L-deprenyl (^11^C-DED)	MAO-B	The deuterium-substituted form of L-deprenyl, radiolabeled with carbon-11, provides high selectivity and sensitivity for imaging MAO-B with a lower trapping rate, mitigating its irreversible binding nature [24].
^18^F-SMBT-1	MAO-B	A highly selective MAO-B tracer, with low nonspecific binding, high entry into the brain, and reversible kinetics [25,26].
^11^C-BU99008	I_2_BS	Reversible binding properties, with good entry into the brain and highly specific and selective binding to I_2_BS [27].

* MAO-B and I_2_BS are overexpressed in the outer mitochondrial membrane of reactive astrocytes.

## Data Availability

Not applicable.
